# Refractory Elevated Intracranial Pressure (ICP) in the Setting of a Traumatic Cerebral Sinus Venous Thrombosis (CSVT)

**DOI:** 10.7759/cureus.17801

**Published:** 2021-09-07

**Authors:** Kunal P Kanakia, Ehsan Saffari, Sabi Shrestha, Viktor Bartanusz, Shaheryar Hafeez

**Affiliations:** 1 Department of Neurology, University of Texas Health Science Center at San Antonio, San Antonio, USA; 2 Department of Neurosurgery, University of Texas Health Science Center at San Antonio, San Antonio, USA

**Keywords:** intracranial pressure, traumatic brain injury, cerebral sinus venous thrombosis, cranial fracture, intracranial hemorrhage

## Abstract

The management of patients with elevated intracranial pressure (ICP) requires a systematic approach. After the failure of tier zero, tier one, and tier two therapies, all potential secondary causes of elevated ICP must be reviewed. Up to 28% of patients with blunt traumatic brain injury (TBI) develop cerebral sinus venous thrombosis (CSVT), among these, patients up to 55% have occlusive thrombi. A literature review revealed a dearth of specific treatment guidelines in this scenario. Here, we present one such case of refractory elevated ICP due to occlusive CSVT secondary to skull fractures. Initial CT venogram (CTV) on admission showed an occlusive CSVT; however, subsequent CTV on the post-trauma day (PTD) 4 and 6 showed non-occlusive thrombi only. The risks of worsening acute TBI-related hemorrhage with systemic anticoagulation versus the benefit of treating an occlusive CSVT are discussed here. In cases of occlusive CSVT with refractory elevated ICP and stable intracranial hemorrhage, the benefit of anticoagulation may outweigh the overall risks of hemorrhage expansion as prolonged uncontrolled ICP elevation is inevitably fatal. In this case, anticoagulation started on PTD 6, led to the resolution of ICP elevation and an excellent outcome for the patient, who was discharged to an acute rehab center, subsequently discharged home with no residual motor deficits, and was able to resume employment. Further prospective trials are necessary to develop guidelines for the management of occlusive CSVT in patients with severe TBI and to determine which patient populations are likely to benefit from early initiation of therapeutic anticoagulation.

## Introduction

The clinical presentation of elevated intracranial pressure (ICP) may be unreliable [1]. Therefore, all patients with severe traumatic brain injury (TBI) require invasive ICP monitoring if the initial Glasgow coma score (GCS) is 8 or less after resuscitation and the initial CT scan shows intracranial hemorrhage (ICH) or concern for obstructive hydrocephalus [2]. Elevated ICP post-TBI has a broad differential and requires a systematic approach. Common differentials include ICH, cerebral edema, obstructive hydrocephalus, meningitis, and encephalitis. Secondary causes include elevated intra-abdominal or intrathoracic pressure and agitation [1]. The Neurocritical Care Society Emergency Neurological Life Support guidelines recommend a tier-based approach (zero to three) to the management of increased ICP, which is defined as a sustained elevation of ICP to more than 22 mmHg for more than five minutes. These approaches are temporizing measures while assessing the underlying etiology of elevated ICP and determining definitive therapy [3]. Failure of tier zero to tier two approaches should prompt a more extensive investigation of underlying causes as mentioned in the case below.

## Case presentation

**Figure 1:**
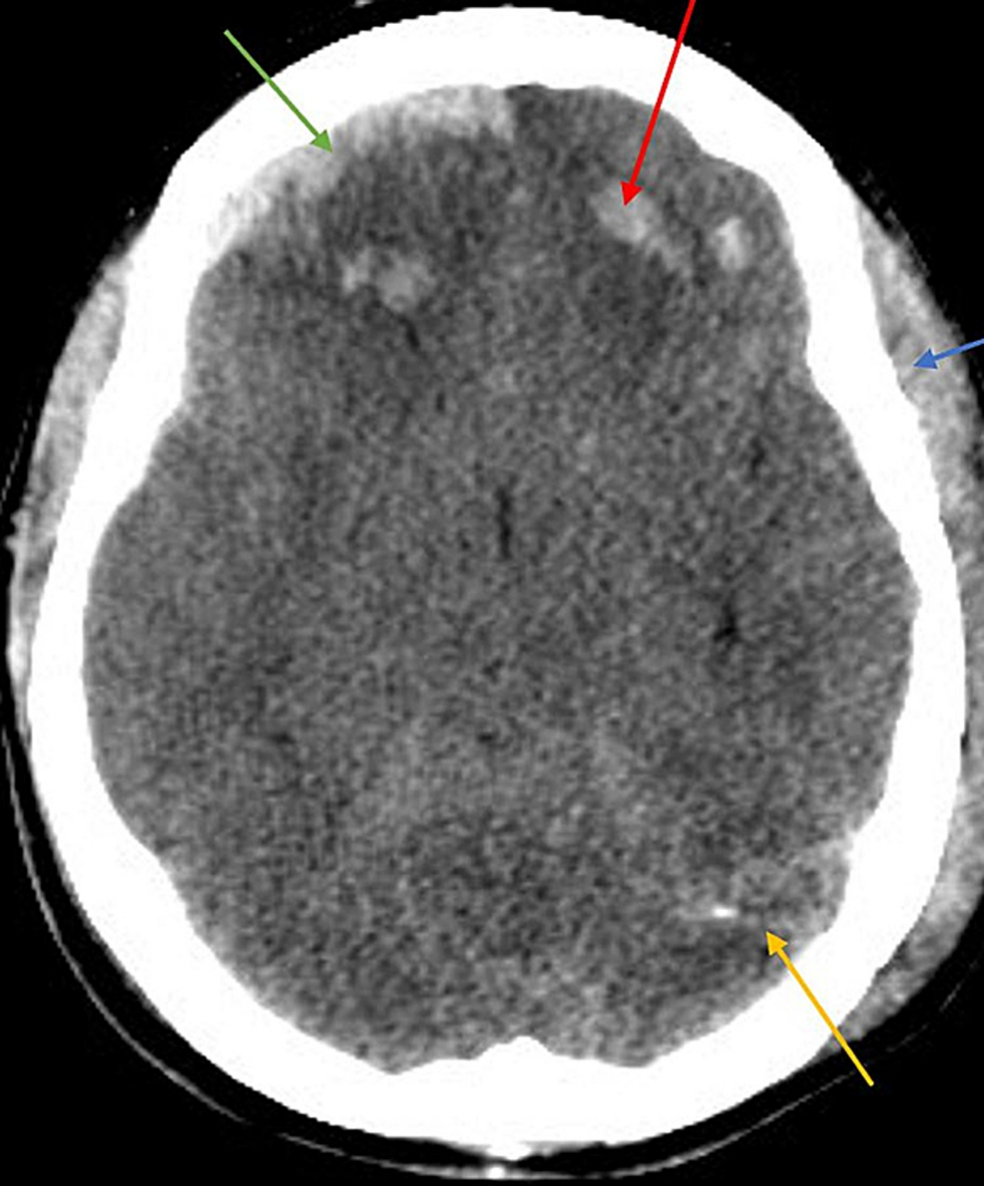
CT head without contrast on admission Green arrow: traumatic right frontal sub-dural hematoma, red arrow: traumatic left frontal hemorrhagic contusion, blue arrow: scalp hematoma; yellow arrow: traumatic sub-arachnoid hemorrhage

**Figure 2:**
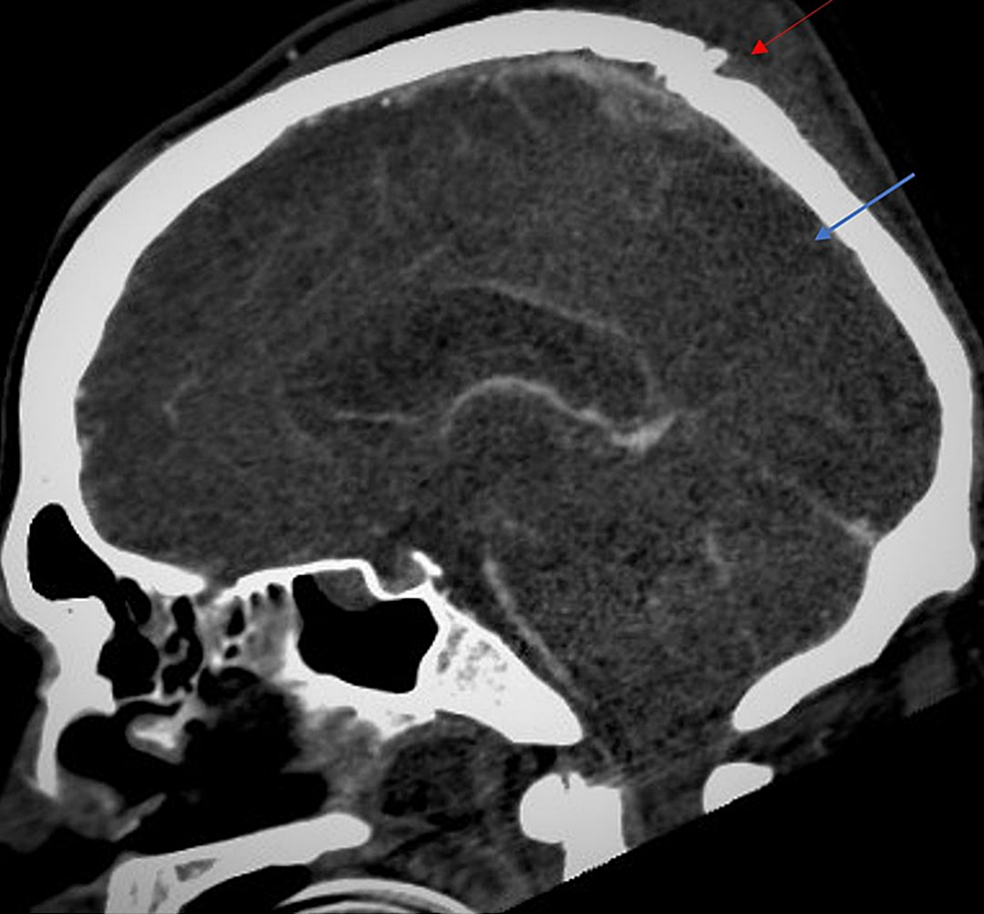
CT venogram on admission Red arrow: depressed skull fracture, blue arrow: non-opacification of contrast medium in posterior one-third of the superior sagittal sinus indicating an occlusive cerebral venous sinus thrombosis

A 42-year-old male with no known past medical history presented to the emergency department (ED) of a level 1 trauma center after an alleged assault. The patient suffered a severe TBI with multiple comminuted skull fractures, left frontal hemorrhagic contusion, right frontal sub-dural hematoma, scalp hematoma, and traumatic subarachnoid hemorrhage (SAH) (Figure 1). GCS was 13 on arrival. Laboratory abnormalities included leukocytosis (14.63 x 10^3^/microlitre; normal 3.40-10.40 x 10^3^/microlitre), elevations in serum creatine kinase (1,192 units/L; normal 24-223 units/L) and lactic acid (3.7 mmol/L; normal 0.5-2.0 mmol/L). CT venogram (CTV) on admission showed an occlusive CSVT of the posterior one-third segment of the superior sagittal sinus (SSS) (Figure 2) and a nonocclusive thrombus in the left transverse sinus. The patient became combative and agitated in the ED leading to haloperidol administration. GCS subsequently dropped to 7; therefore, the patient was intubated for airway protection and admitted to the neurosurgical intensive care unit (NSICU).

**Figure 3:**
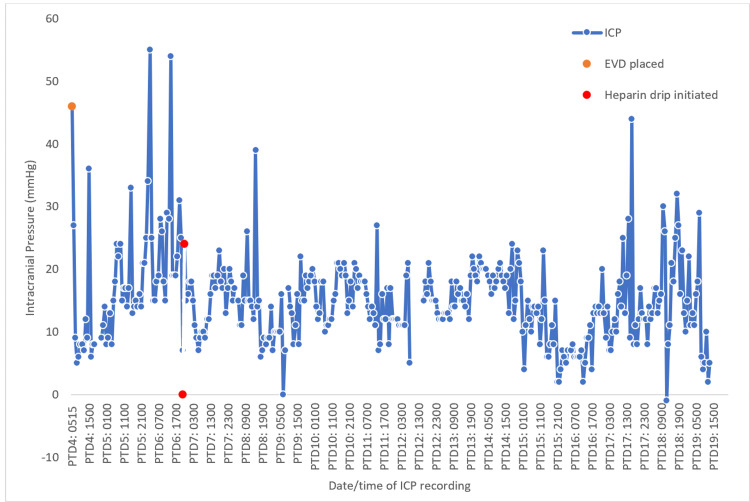
Graph showing patient’s intracranial pressure readings in mmHg plotted against time ICP: intracranial pressure, EVD: external ventriculostomy drain, PTD: post-trauma day *Interruption in ICP values at PTD 12 was due to an error in the medical record software leading to loss of data

Over the next 48 hours, GCS fluctuated from 7 to 10. Empiric treatment was started including therapeutic hypernatremia with a sodium goal of 150-155 MeQ/L. Sedation with fentanyl and midazolam infusions. CTV on post-trauma day (PTD) 3 showed no occlusive cerebral sinus venous thrombosis (CSVT), only a non-occlusive left transverse sinus thrombosis. On PTD 4, the patient developed bilateral dilated non-reactive pupils. Mannitol and 23.4% hypertonic saline were administered. Imaging showed stable ICHs with no evidence of hydrocephalus. An external ventriculostomy drain (EVD) was placed, showing an ICP of 48 mmHg. 20 mL cerebrospinal fluid (CSF) was drained which transiently normalized the ICP. On PTD 6, ICP increased to 40 mmHg. 250 mL bolus of 3% saline was given, followed by a 24-hour infusion. A bolus of ketamine and rocuronium was also given. Sodium goals were increased to 155-160 MeQ/L. Failure of tier zero to tier two therapies over the next six days led to concern for the propagation of the CSVT or new occlusive CSVT. ICP fluctuated between 19 and 54 mmHg over the next 24 hours (Figure 3).

**Figure 4:**
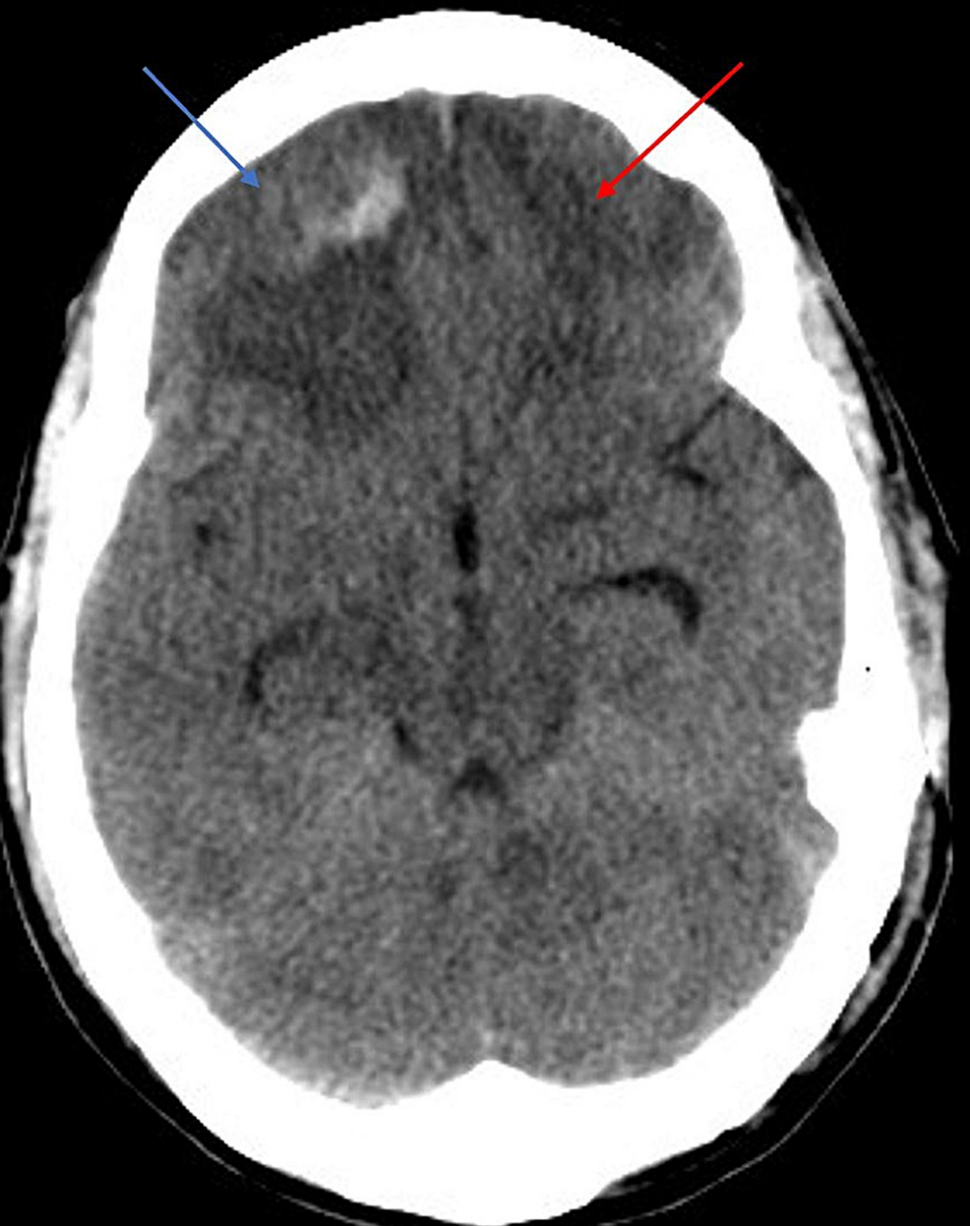
CT head without contrast at discharge showing resolving traumatic intracranial hemorrhages Blue arrow: resolving right frontal sub-dural hematoma, red arrow: resolving left frontal hemorrhagic contusion

Repeat CTV on PTD 6 was the same as PTD 3. However, due to high clinical suspicion, a heparin drip was initiated after confirming stable ICHs. ICP started resolving to 11-22 mmHg with intermittent spikes to 44 mmHg coinciding with agitation (Figure 3). Cisatracurium and pentobarbital infusions were started to prevent further spikes of ICP. Diagnostic cerebral angiography on PTD 9 showed non-occlusive thrombi in straight and right transverse sinus with severe stenosis in the mid and posterior SSS. ICP continued to trend within normal limits. On PTD 16, the patient was extubated; by PTD 19, heparin infusion was discontinued and the patient was transitioned to a therapeutic dose of low molecular weight heparin. EVD was removed on PTD 21. The patient was downgraded from the NSICU on PTD 23, apixaban was started and he was discharged on PTD 39 for neurorehabilitation with a CT head at discharge (Figure 4) showing resolution of the ICHs seen in Figure 1.

The patient was discharged home after three weeks of inpatient rehabilitation therapy with no motor deficits. MR venogram at six weeks post-discharge showed no residual CSVT. Seven weeks after hospital discharge patient had no motor deficits but reported persistent memory loss about the event. Overall, he was happy with his recovery. Twelve weeks post-discharge apixaban was stopped and low dose aspirin was started. The patient resumed previous employment 12 weeks after hospital discharge. He currently follows up with a Physical Medicine and Rehabilitation specialist and has not had any re-admissions since.

## Discussion

The incidence of post-traumatic CSVT is 28% with blunt TBI involving skull fractures crossing the suture lines, among them, 55% have occlusive thrombi [4]. Typical compensation mechanisms for elevated ICP include CSF and venous displacement. In the above case, CSVT prevents this compensation, thereby leading to refractory ICP elevation. In non-traumatic CSVT cases, therapeutic anticoagulation is started immediately, despite the presence of acute ICH or stroke, because further clot propagation will worsen the hemorrhage or stroke. Therefore, the risk of hemorrhagic expansion with therapeutic anticoagulation is outweighed by the benefit of decreasing clot propagation [5]. In contrast, up to one-third of patients with severe TBI have contusion blossoming and hematoma expansion due to coagulopathy, hypertension, blood vessel shearing, damaged necrotic tissue, and secondary inflammatory injury. It would be extremely harmful if any type of anticoagulation is initiated in this phase of acute TBI due to the risk of hemorrhagic expansion or contusion blossoming [6]. However, delay of CSVT diagnosis and treatment can lead to an increased need for surgery and a higher risk of mortality [7].

Management

There are no specific guidelines addressing the use of systemic anticoagulation in traumatic CSVT. The optimal timing of initiation of systemic anticoagulation is challenging due to a lack of clear guidelines and potentially devastating consequences of hemorrhage expansion. Some authors recommend initial expectant management especially in children and when ICP is stable [8], while others recommend early therapeutic anticoagulation within 48 hours of injury if ICHs are stable [9]. The duration of anticoagulation for traumatic CSVT is also not well defined. Hersch et al. described a case series where three months of systemic anticoagulation led to the resolution of CSVT in 50% of cases. Long-term systemic anticoagulation, however, exposes patients to the risk of symptomatic hemorrhages; the same case report found a 14% incidence of both intracranial and extracranial hemorrhages with a mortality rate of 4.5% [9]. There are however well-defined guidelines from the 2017 European Stroke organization for the duration of anticoagulation for non-traumatic CSVT, which could provide some guidance. They recommend a therapeutic dosage of heparin to all patients with acute non-traumatic CSVT, even in the presence of intracerebral hemorrhage. Additionally, the AHA/ASA guidelines recommend anticoagulation for 3-6 months in provoked non-traumatic CSVT, 6-12 months in unprovoked non-traumatic CSVT, and potentially lifelong in recurrent non-traumatic CSVT [10]. In cases where the ICHs are stable, intravenous thrombolytic therapy [7], and mechanical thrombectomy are alternative but effective therapeutic options [11].

## Conclusions

The risks of worsening ICH with systemic anticoagulation versus the benefit of treating an occlusive CSVT must be considered by all parties involved. In cases of occlusive traumatic venous sinus thrombosis with refractory elevated ICP and stable ich, the benefit of anticoagulation may outweigh the overall risks of hemorrhage expansion, as prolonged uncontrolled ICP elevation is inevitably fatal. Optimal timing systemic anticoagulation initiation and total duration of treatment remain a challenge. In the above case, systemic anticoagulation with a therapeutic heparin infusion was started on PTD 6 and continued for two weeks, after which the patient was initially transitioned to low molecular weight heparin, followed by oral anticoagulation with apixaban, for a total anticoagulation duration of 12 weeks. This led to the resolution of ICP elevation and an excellent outcome for the patient. Further prospective trials are necessary to develop guidelines for the management of occlusive venous sinus thrombosis in patients with TBI and to determine which patient populations are likely to benefit from early initiation of therapeutic anticoagulation, the optimal time to initiate anticoagulation, and the optimal duration of anticoagulation.
